# Triglyceride-Glucose Index and Atherogenic Index as Alternative Biomarkers for Glycemic Control in Type 2 Diabetes Mellitus

**DOI:** 10.7759/cureus.81550

**Published:** 2025-03-31

**Authors:** Margit Gajjar, Mritunjay K Mishra, Tejas J Shah, Sajidali S Saiyad, Jay Prakash S Rajput

**Affiliations:** 1 Biochemistry, Gujarat Medical Education and Research Society (GMERS) Medical College, Rajpipla, IND; 2 Physiology, Pacific Medical College and Hospital, Udaipur, IND

**Keywords:** atherogenic index, glycemic control, predictive biomarkers, triglyceride glucose index, type 2 diabetes mellitus

## Abstract

Background: The triglyceride-glucose (TyG) index and the atherogenic index (AI) are emerging biomarkers that have been gaining attention in diabetes management as alternatives for assessing glycemic control in clinical settings. Since direct measurement of insulin resistance is impractical in routine care, it offers a more accessible alternative with potential broad clinical application. The TyG index and AI can serve as useful substitute biomarkers and could help in the management of glycemic control in type 2 diabetes mellitus (T2DM).

Methodology: A cross-sectional study was conducted with 200 T2DM participants, divided into two groups: 100 with good glycemic control (<7.0%) and 100 with poor control (≥7.0%), based on HbA1c levels. Lipid profile and HbA1c were measured using Chem-7 (Erba Mannheim, India) and Insta Check semi-auto analyzers. TyG index, TyG-body mass index (BMI), and TyG-waist circumference (WC) were calculated using standard formulas. Statistical analysis was performed by using SPSS 20.0 (IBM Corp., Armonk, New York, US), with p < 0.05 considered statistically significant.

Results: Among 200 subjects, 63% were men and 37% were women. The mean age for type 2 diabetes subjects is 61.24 ± 7.25 years. The mean level of the TyG index in poor glycemic control is significantly higher (30.36 ± 5.51, p < 0.001) than that of good glycemic control (4.06 ± 0.05, p < 0.001). A significant positive correlation was observed between the TyG index and poor glycemic control. The TyG index has good predictive ability in poor glycemic control (area under the curve (AUC): 0.88; 95% confidence interval (CI): 0.83-0.92). The TyG optimal cutoff is ≥5.22 with 78.22% sensitivity and 94.06% specificity.

Conclusion: The TyG index shows a significant correlation with glycemic control and could serve as a valuable supportive marker for T2DM, particularly in smaller clinical settings.

## Introduction

Diabetes mellitus is a serious health concern. Globally, type 2 diabetes mellitus (T2DM) prevalence is on the rise due to aging, physical inactivity, urbanization, and the increasing prevalence of obesity, which leads to an economic burden. As per the International Diabetes Federation (IDF) report, the worldwide prevalence of T2DM is predicted to rise from 8.8% to 10.3% by 2045 [1]. Approximately 40 million T2DM cases, with a good majority across the country, are due to unawareness of diseases and different comorbid factors such as aging, high body mass index (BMI), and greater exposure to lifestyle-related risk factors [2]. With changes in the diabetes risk with aging, early detection and intervention might be helpful to prevent serious disease complications and reduce costs in the healthcare system [3]. Insulin sensitivity and effective glycemic control are essential components to decrease the risk for progression of type 2 diabetes. Currently, glycosylated hemoglobin (HbA1c) and the homeostasis model assessment of insulin resistance (HOMA-IR) are frequently being used to measure long-term blood glucose levels and insulin resistance (IR). HbA1c along with lipid parameters are being used to predict macro- and microvascular complications of T2DM [4,5]. Macro- and microvascular complications in diabetes consisting of increased triglycerides (TGs), reduced high-density lipoprotein cholesterol (HDL-C), and increased low-density lipoprotein cholesterol (LDL-C) levels are considered consequences of hyperinsulinemia and glycemic control. Among the lipid indices, the TG/HDL-C ratio is one of the known atherogenic indices for metabolic syndrome and cardiovascular risk. Numerous previous literatures reported that higher levels of the TG/HDL-C ratio have an impactful association with endothelial dysfunction and also were an indicator of IR [5,6]. Lee et al. [7] and Sánchez-Escudero et al. [8] have reported that the triglyceride-glucose (TyG) index exhibited a significant correlation with HOMA-IR and was linked to a higher prevalence of metabolic syndrome and suggested that the TyG index could be an identifying tool for IR and related metabolic disorders.

The TyG index is a product of TG and glucose and measures both individual glycemic control and cardiovascular status [9]. An inexpensive and simpler substitute biomarker is being recommended for IR, in relation to the HOMA-IR [10]. Numerous researchers have reported a good link between glycemic control and the TyG index, suggesting its significant role in T2DM patient assessment and management [11,12]. A number of studies represented longitudinal determination without population variability to get a relation between the TyG index and glycemic control. An interpretation of the TyG index relation with diabetes with changes in glycemic control and population variability is required for long-term outcome and demographic application [11]. Looking into the above fact, the current study intends to assess the potential role of the TyG index and atherogenic index and its relationship with HbA1c and evaluate their predictive utility for glycemic control with T2DM.

## Materials and methods

A prospective, analytical, cross-sectional study was conducted in the Department of Biochemistry, Gujarat Medical Education Research Society (GMERS) Medical College, Vadnagar, Gujarat, India, within the duration of April to May 2022. A total of 200 T2DM patients with an age group of 40-65 years who visited the OPD of GMERS, Vadnagar, were enrolled as per the minimum standard of the American Diabetes Association (ADA) guidelines [13]. The study has been approved by the Institutional Ethical Committee (Human Research) of GMERS Vadnagar, Gujarat (Approval No. GMERS/MCV/IEC/(HR)/Approval/5619/2022).

The total sample size has been calculated by using the prevalence of T2DM in Gujarat [14], i.e., 8% with 10% allowable error at a 95% confidence interval (CI), to ensure the statistical power of the study with the following formula:

n = 4PQ/L^2^

where n = total no. of sample, P = prevalence of diabetes in Gujarat (8%), and L^2^ = allowable error, i.e., 5%.

As per the statistical calculation, the number of total samples to be collected is 117, but in response to obtaining a precise result while comparing the present study, we have considered and enrolled a total of 200 T2DM patients as per the standard diagnostic criteria. Based on their HbA1c levels, enrolled patients were categorized equally into two groups. HbA1c level < 7.0% is considered "good glycemic control" and ≥7.0% as "poor glycemic control." Subjects who were diagnosed with type 1 diabetes or other coexistence of serious conditions such as liver diseases, renal diseases, or any other vulnerable diseases and subjects on steroidal medication were excluded from this study. After a brief explanation of the study plan, written and verbal consent was obtained from each participant. Participants provided sociodemographic and clinical data, including anthropometric measurements such as weight, height, and waist circumference (WC).

Sample collection and analysis

A total of 5 mL of blood samples (with a minimum of eight hours of fasting) was collected using an aseptic technique with prior instruction. Collected blood was dispensed into three different vacutainers, i.e., plain for lipid parameters, sodium fluoride for blood glucose, and ethylenediaminetetraacetic acid (EDTA) for HbA1c measurement. The blood in the plain and fluoride vacutainers was centrifuged at 5,000 rpm for 10 minutes to get serum and plasma samples. The measurement of blood glucose and lipid profile was done by colorimetric assays using a Chem-7 semiautomatic analyzer (Erba Mannheim) from India. HbA1c was measured by the nephelometry method in the Mispa i3 automated cartridge-based analyzer from India. The serum level of LDL-C was calculated by using Friedewald’s formula: LDL-C (mg/dL) = total cholesterol (TC) (mg/dL) − HDL-C (mg/dL) − TG (mg/dL)/5 [15].

TyG index calculation

The TyG index is determined by taking the logarithm of the product of two metabolic markers, fasting TGs and fasting glucose (FG), and then dividing by two, i.e., TyG index = Log (fasting TGs (mg/dL) × FG (mg/dL))/2 [16]. Lipid ratio, i.e., TG/HDL ratio, is calculated by TG (mg/dL)/HDL-C (mg/dL). TyG-WC was calculated by the multiplication of TyG with WC (TyG index × WC), and TyG-BMI was calculated by the multiplication of TyG with BMI (TyG index × BMI) [16].

Statistical analysis

Study Tools

The data were collected using a case record form (CRF). The CRF includes diagnosis details, sociodemographic information, clinical data, anthropometric measurements (weight, height, and WC), and serum biochemical marker values. Data were analyzed using SPSS version 20 for Windows (IBM Corp., Armonk, New York, US) after being entered into Microsoft Office Excel (Microsoft Corp., Redmond, WA, US). Descriptive statistics were reported using mean, standard deviation, and standard error of the mean. The Shapiro-Wilk test was used to ensure that the data had a normal distribution. A comparison of categorical variables between poor and good glycemic control was done by using a chi-squared test. The serum biochemical markers of the two groups were compared using an independent sample t-test (two-tailed). Pearson correlation analysis was used to examine the relationship between variables and indices. The receiver operating characteristic (ROC) curve and optimal cut-off values were determined at 95% CI. A p-value < 0.05 was considered statistically significant.

## Results

**Figure 1:**
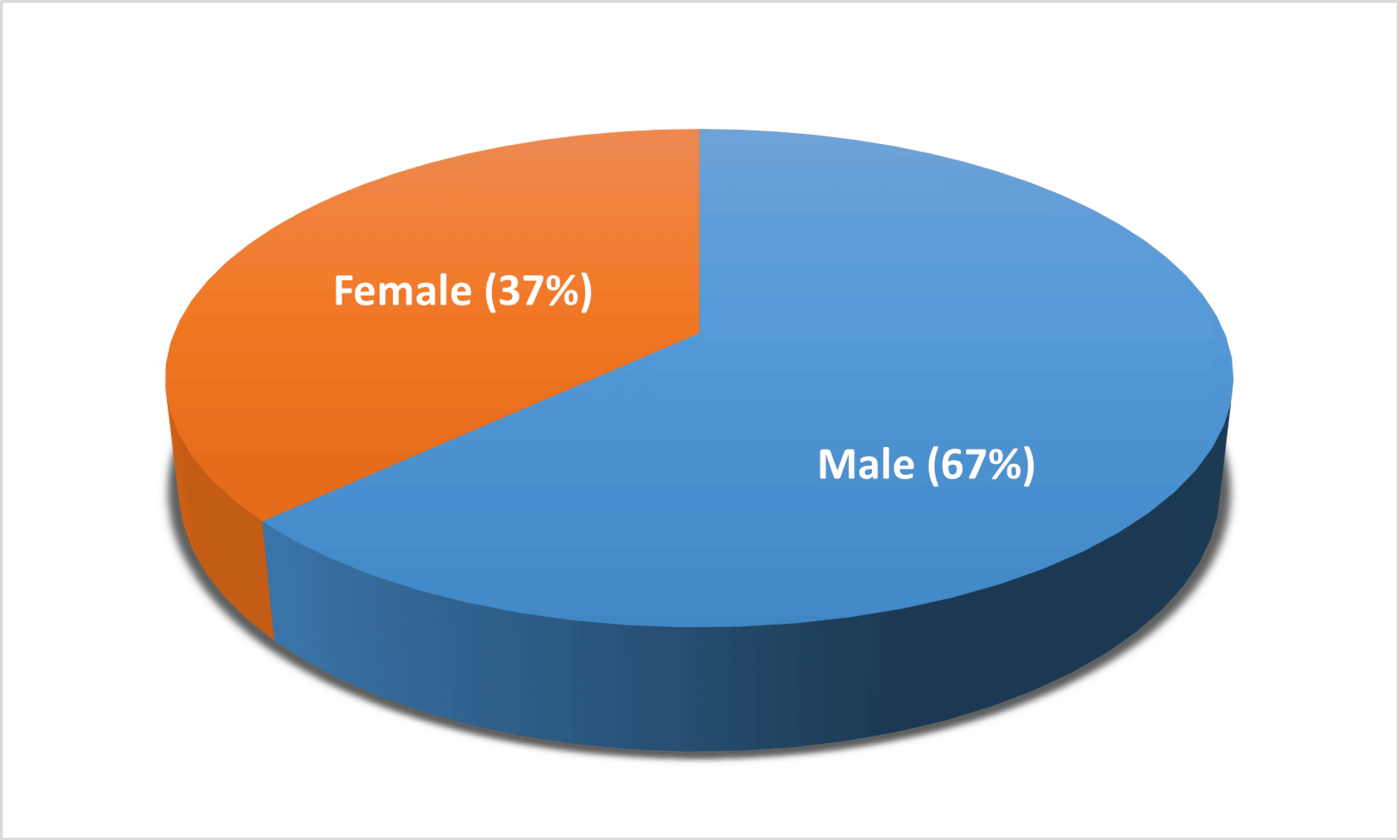
Gender distribution of study participants (N = 200)

**Figure 2:**
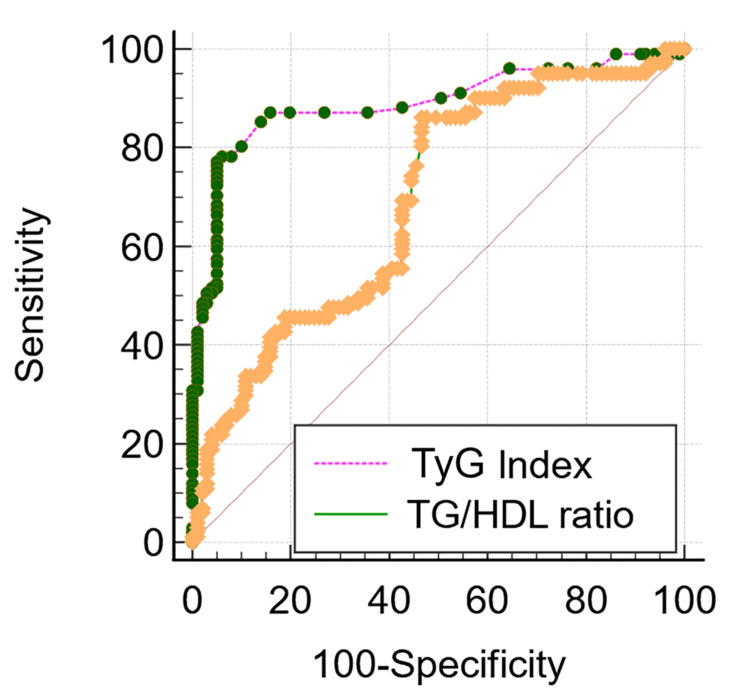
Receiver operating characteristic (ROC) curve analysis for the TyG index and atherogenic index in predicting poor glycemic control in T2DM T2DM: type 2 diabetes mellitus; TyG: triglyceride-glucose; TG/HDL ratio: triglyceride-to-high-density-lipoprotein ratio

Among a total of 200 subjects with a confirmed diagnosis of T2DM, 50% were in <7.0% and 50% were in ≥7.0%. The present study has 63% men and 37% women (Figure 1). The participant's mean age is 61.24 ± 7.25 years. However, the mean age for good glycemic control is 57.47 ± 11.84 years, and the mean age for poor glycemic control is 65.13 ± 2.67 years, respectively. Table 1 depicts the baseline anthropometric characteristics for individuals with good and poor glycemic control. Table 2 comprises participants’ habits, such as smoking, non-smoking, and alcohol consumption, in poor and good glycemic controls. Table 3 represents a comparison of biomarkers among poor and good glycemic control. The study compares the biochemical parameters and metabolic indexes of diabetic patients with good and poor glycemic control. Fasting blood glucose (144.05 ± 45.70, p < 0.001), HbA1c (8.70 ± 1.92, p < 0.001), TG/HDL (8.34 ± 3.65, p < 0.001), and TyG index (30.36 ± 5.51, p < 0.001) ratios are significantly higher in poor glycemic control than in good glycemic control. Table 4 depicts an association of the TyG index and TG/HDL ratio with other biochemical markers, which was significantly associated. Table 5 and Figure 2 show ROC curve analysis with higher area under the curve (AUC) and specificity of the TyG index than the atherogenic index.

**Table 1:** Baseline characteristics and demographic data in T2DM Unpaired t-test p-value: **p < 0.001, *p < 0.05, and ^#^p > 0.05 (not significant) T2DM: type 2 diabetes mellitus; BMI: body mass index; WC: waist circumference; SD: standard deviation; SEM: standard error of the mean

Variables	Good glycemic control (<7.0%) (N = 100)		Poor glycemic control (≥7.0%) (N = 100)		p-value (unpaired t-test)
	Mean ± SD	SEM	Mean ± SD	SEM	
Age (years)	57.47 ± 11.44	1.18	65.13 ± 2.67	0.27	0.77^#^ (-6.52)
Height (cm)	176 ± 6.77	0.67	175 ± 5.22	0.52	0.24^#^ (1.16)
Weight (kg)	78.4 ± 2.45	0.24	74.7 ± 3.88	0.50	0.001** (8.06)
BMI (kg/cm²)	25.22 ± 6.77	0.67	27.4 ± 5.02	0.50	0.01* (-2.58)
WC (cm)	84.75 ± 8.53	0.85	98.94 ± 13.46	1.34	0.001** (-8.90)

**Table 2:** Baseline characteristics of categorical variables in T2DM Chi-squared test p-value: **p < 0.001 T2DM: type 2 diabetes mellitus

Categorical variables	Good glycemic control (<7.0%) (N = 100)	Poor glycemic control (≥7.0%) (N = 100)
Smoker (%)	40 (40%)	67 (67%)
Non-smoker (%)	60 (60%)	33 (33%)
Chi square (χ²) (p-value)	14.65 (0.001**)	
Alcoholic (%)	37 (37%)	63 (63%)
Non-alcoholic (%)	73 (73%)	27 (27%)
Chi square (χ²) (p-value)	26.18 (0.001**)	

**Table 3:** Analytical comparison of biomarkers based on glycemic control in T2DM Unpaired t-test p-value: **p < 0.001, *p < 0.05, and ^#^p > 0.05 (not significant) SD: standard deviation; SEM: standard error of the mean; HbA1c: glycosylated hemoglobin; FBS: fasting blood sugar; 2-h PG: 2-hour post glucose; TG: triglyceride; HDL-C: high-density lipoprotein cholesterol; LDL-C: low-density lipoprotein cholesterol; TG/HDL ratio: triglyceride-to-high-density-lipoprotein ratio; TyG index: triglyceride-glucose index; TyG-WC: triglyceride-glucose-waist circumference; TyG-BMI: triglyceride-glucose-body mass index; T2DM: type 2 diabetes mellitus

Parameters	Good glycemic control (<7.0%) (N = 100)		Poor glycemic control (≥7.0%) (N = 100)		t-test	p-value
	Mean ± SD	SEM	Mean ± SD	SEM		
HbA1c (%)	6.22 ± 1.18	0.12	8.57 ± 1.90	0.19	10.50	≤0.001**
FBS (mg/dL)	120.33 ± 8.39	0.84	144.05 ± 45.70	4.55	5.10	≤0.001**
2-h PG (mg/dL)	233.16 ± 67.86	6.75	260.48 ± 63.19	6.29	2.94	≤0.001**
T. cholesterol (mg/dL)	193.93 ± 65.48	6.52	195.66 ± 15.66	1.56	0.25	>0.05^#^
TG (mg/dL)	191.68 ± 15.30	1.52	231.72 ± 70.24	6.99	5.57	≤0.001**
HDL-C (mg/dL)	36.08 ± 12.97	1.29	29.80 ± 6.14	0.61	-4.37	≤0.001**
LDL-C (mg/dL)	139.08 ± 40.47	4.03	128.70 ± 19.92	1.53	-2.30	≤0.01*
TG/HDL ratio	6.23 ± 2.86	0.29	8.34 ± 3.65	0.36	4.55	≤0.001**
TyG index	4.22 ± 0.77	0.08	7.75 ± 3.98	0.40	8.70	≤0.001**
TyG-WC indices	356.85 ± 68.31	6.80	759.85 ± 393.44	39.93	10.09	≤0.001**
TyG-BMI indices	106.87 ± 37.09	3.69	234.13 ± 127.11	12.65	9.61	≤0.001**

**Table 4:** Pearson correlation analysis of TyG indices and atherogenic index with other biochemical markers in T2DM Unpaired t-test p-value: **p < 0.001, *p < 0.05 TyG: triglyceride-glucose; FBS: fasting blood sugar; TG: triglyceride; HDL-C: high-density lipoprotein cholesterol; LDL-C: low-density lipoprotein cholesterol; TyG-WC: triglyceride-glucose-waist circumference; TyG-BMI: triglyceride-glucose-body mass index; T2DM: type 2 diabetes mellitus; 2-h PG: 2-hour post glucose; TG/HDL ratio: triglyceride-to-high-density-lipoprotein ratio; HbA1c: glycosylated hemoglobin

Variables	Good glycemic control (<7.0%) (N = 100)		Poor glycemic control (≥7.0%) (N = 100)	
	TyG indices (r-value)	TG/HDL ratio (r-value)	TyG indices (r-value)	TG/HDL ratio (r-value)
FBS (mg/dL)	0.79**	0.22	0.85**	0.3
2-h PG (mg/dL)	0.52**	0.35*	0.70**	0.33
HbA1c	0.30*	0.12*	0.11	0.01
T. cholesterol (mg/dL)	0.55**	0.40*	0.35	0.04
TG (mg/dL)	0.73**	0.37*	0.90**	0.82**
HDL-C (mg/dL)	-0.24*	-0.89**	-0.20	-0.75**
LDL-C (mg/dL)	0.51**	0.43*	0.19	0.02
TyG-BMI indices	0.44*	0.18	0.85**	0.20
TyG-WC indices	0.40*	0.16	0.84**	0.37

**Table 5:** AUC, optimal cutoff, sensitivity, and specificity of TyG index and atherogenic index in T2DM p-value: **p < 0.001 AUC: area under the curve; CI: confidence interval; PPV: positive predictive value; NPV: negative predictive value: T2DM: type 2 diabetes mellitus; TG/HDL ratio: triglyceride-to-high-density-lipoprotein ratio; TyG: triglyceride-glucose

Variables	AUC	95% CI		Youden index (J)	Cutoff value	Sensitivity (%)	Specificity (%)	PPV	NPV	p-value
		Upper limit	Lower limit							
TyG index	0.88	0.83	0.92	0.72	4.16	78.22	94.06	92.13	81.10	<0.001**
TG/HDL ratio	0.62	0.62	0.75	0.39	5.22	86.14	53.47	64.66	79.10	<0.001**

## Discussion

The present study has included a total of 200 T2DM patients, out of which 63% were men and 37% were women. Out of 200, 100 T2DM patients have poor glycemic control, i.e., ≥7.0%, and 100 T2DM patients have good glycemic control, i.e., <7.0%. Currently, hyperglycemia, a characteristic of metabolic disorders, has become one of the top-notch reasons for mortality worldwide [17]. Presently, markers being used to diagnose and monitor the diseases are fasting and post-absorptive plasma glucose, which requires 8-10 hours of fasting for sample collection, oral glucose tolerance, and HbA1c, which reproduces long-term glycemic control. The levels of HbA1c might change due to glycemia or erythrocyte turnover rate, making it an unreliable marker for patients who have diseases related to red blood cells (RBCs) [18]. On the other hand, it can be a time-consuming procedure, and also, it may not be easily available in primary care. Due to these disadvantages, the present study has analyzed an alternative and supplementary marker, which is the TyG index. The TyG index is a reasonable and non-insulin-dependent biomarker that requires TG and fasting plasma glucose estimation, which is commonly available and regularly done in routine laboratories. To contribute to this gap, we have focused on the Indian Gujarat state tribal area population with a minimum primary care setting [18].

The assessment of risk factors linked to poor glycemic control and their variability was explained by Dinavari et al. [19], who demonstrated significant differences between T2DM patients with good and poor glycemic control in factors such as family history, habits, socioeconomic status, hypertension, diabetes, sleep duration, and certain chronic diseases. The current study focuses on habits like smoking and alcohol consumption, finding a higher prevalence of smokers and alcoholics in the group with poor glycemic control compared to those with good glycemic control.

The dyslipidemic phenomenon in diabetes can be attributed to alterations in plasma lipoprotein levels in diabetic patients during both fasting and postprandial states, which are influenced by IR and the hyperglycemic state. In the postprandial state, fatty acids and cholesterol derived from food get absorbed and combined into TGs and cholesteryl esters, which are then packaged into chylomicrons, which further trigger lipolysis. IR can be an independent cause of abnormal lipid parameters in a hyperglycemic state by increasing the production of ApoC-III (LDL-C) and decreasing the production of Apo A-I (HDL-C). This imbalance results in enhanced lipid uptake by the endothelium [20].

According to the ADA guidelines, glycemic control should be assessed based on self-monitoring of blood glucose (SMBG) levels and HbA1c. HbA1c levels of less than 7.0 are clinically considered good glycemic control and are regarded as the gold standard for assessing long-term blood sugar regulation [13,18].

Kumar and Sandhya [21] have conducted a study and showed a higher prevalence of coronary artery disease (CAD) in patients with poor glycemic control. The present study has also demonstrated the lipid components and observed a similar pattern.

Changes in dyslipidemia in T2DM with poor glycemic control can be exemplified by the atherogenic index, i.e., the TG/HDL ratio, and decreased levels of HDL-C. In diabetes, decreased insulin sensitivity, low HDL-C, and hypertriglyceridemia result in highly impacted blood levels of cholesteryl ester transfer protein (CETP), which facilitates the transport of cholesterol ester and triacylglycerol between HDL-C and LDL-C particles [21,22]. This results in elevated levels of LDLs. This is one of the trademarks of diabetic-linked cardiovascular risk. However, the estimation of LDL-C is an incommodious procedure; the determination of the TG/HDL ratio indirectly reflects LDL size, which is comparable to the actual evaluation of small dense LDL particles [23]. We found a significantly elevated level of TG/HDL-C ratio in poor glycemic control than in good glycemic control. Babic et al. [24] showed that the TG/HDL-C ratio was found to be a useful marker in the prediction of glycemic control in T2DM.

The present study has compared the TyG index and TyG-derived indices such as the TyG-WC index and TyG-BMI and has observed significantly higher levels of poor glycemic control than that of good glycemic control of T2DM. This could be due to the increased susceptibility of beta cells to glucotoxicity and lipotoxicity, which leads to an accumulation of reactive oxygen species (ROS), causing damage to the beta cells, which ultimately affect glucose metabolism [25].

Selviet al. [26] have conducted a study with similar criteria and found similar patterns. In comparison to other indexes, the TyG index was investigated to be a good prognostic indicator for glycemic control. The current findings of this study are supported by Hameed [10] and Timalsina et al. [11]. They have also found that the index was significantly elevated in diabetics with poor glycemic control.

In addition to that, the present study also did a correlation analysis and has shown a strong inverse association between TG/HDL-C and HDL-C in both poor and good glycemic control. In this study, we have found a strong association in the TG/HDL-C ratio and TyG index with other glycemic control and lipid parameters and established a significant association. Combined with the one study conducted by Babic et al. [24] on 113 patients, it showed increased levels of the TyG index with poor glycemic control and suggested the utility of the TyG index in obese and overweight individuals among those with T2DM. Similarly, Hameed [10], on 229 subjects, reported that TyG and TyG-derived indices are significantly increased in poor glycemic control. Lee et al. [27] concluded that the TyG index could be used as diagnostic criteria for identifying individuals who are metabolically obese but at normal weight.

Moreover, the present study also reported the predictive ability of the TyG index and atherogenic index, i.e., the TG/HDL ratio. The TyG index has a higher AUC (0.88) with a cutoff value of 4.16 than the TG/HDL ratio (0.62). However, the TG/HDL have a lesser AUC (0.62) with a cutoff of 5.22 and have more sensitivity (86.14%) than the TyG index (78.22%) but have less specificity (53.47%) than the TyG index (94.06%) at 95% CI. In alignment with the present results, the study conducted by Timalsina et al. [11] by evaluating the TyG index and the TyG-derived index has reported a higher AUC for the TyG index with higher sensitivity and specificity. Similarly, the study conducted by Rathore et al. [28] observed a slightly higher AUC (0.90) for the TyG index with a cutoff of 5.18, a sensitivity of 79.2%, and a specificity of 88.8%. Flake et al. [29] identified a cutoff of >8.4 with a sensitivity of 92.5% and specificity of 47.1%. The TyG index combines FG and TG levels, reflecting two aspects of IR. It captures hepatic IR through FG and adipose tissue resistance via TGs. Since IR is a key factor in the development of type 2 diabetes, this could explain why the TyG index is a strong predictor for assessing glycemic control. In line with previous research, our study discovered that the TyG index was a useful screening tool for predicting glycemic control and IR in T2DM [30]. Our study has a few strengths, such as clinical measurement instead of self-reported outcomes, captured lifestyle factors (i.e., smoking and liquor consumption), and followed high-standard laboratory procedures.

Limitation of the study

The present study is a single-centric, small-scale observational study, and future research with a larger sample size in a general population would be beneficial to confirm the clinical utility and applicability of the TyG index across diverse populations. However, potential confounding factors that could explain the link between poor glycemic control and dyslipidemia were not considered. Patients with inadequate glycemic control and poor treatment adherence may also be less likely to engage in preventive practices, such as regular exercise and maintaining a healthy diet.

## Conclusions

The TyG index and atherogenic index are highly associated with the risk of T2DM. Additionally, a higher TyG/HDL-C ratio could serve as a useful preliminary marker in clinical practice to predict dyslipidemic changes or cardiovascular risk in diabetes patients. The TyG index could also be a potential alternative and cost-effective marker for assessing glycemic control in resource-limited settings or primary care in tribal areas. The TyG index could become a valuable tool for monitoring disease progression and optimizing patient care in type 2 diabetes.
